# Salmon fibrinogen and chitosan scaffold for tissue engineering: in vitro and in vivo evaluation

**DOI:** 10.1007/s10856-018-6192-8

**Published:** 2018-11-30

**Authors:** Ivo Laidmäe, Kaspars Ērglis, Andrejs Cēbers, Paul A. Janmey, Raivo Uibo

**Affiliations:** 1 Department of Immunology, Institute of Biomedicine and Translational Medicine, University of Tartu, 50411 Tartu, Estonia; 2 Institute of Pharmacy, University of Tartu, 50411 Tartu, Estonia; 3 Faculty of Physics, Mathematics and Optometry, University of Latvia, Riga, LV-1002 Latvia; 4 Institute for Medicine and Engineering and Center for Engineering Mechanobiology, University of Pennsylvania, Philadelphia, PA 19104 USA

## Abstract

3D fibrous scaffolds have received much recent attention in regenerative medicine. Use of fibrous scaffolds has shown promising results in tissue engineering and wound healing. Here we report the development and properties of a novel fibrous scaffold that is useful for promoting wound healing. A scaffold made of salmon fibrinogen and chitosan is produced by electrospinning, resulting in a biocompatible material mimicking the structure of the native extracellular matrix (ECM) with suitable biochemical and mechanical properties. The scaffold is produced without the need for enzymes, in particular thrombin, but is fully compatible with their addition if needed. Human dermal fibroblasts cultured on this scaffold showed progressive proliferation for 14 days. Split-thickness experimental skin wounds treated and untreated were compared in a 10-day follow-up period. Wound healing was more effective using the fibrinogen-chitosan scaffold than in untreated wounds. This scaffold could be applicable in various medical purposes including surgery, tissue regeneration, burns, traumatic injuries, and 3D cell culture platforms.

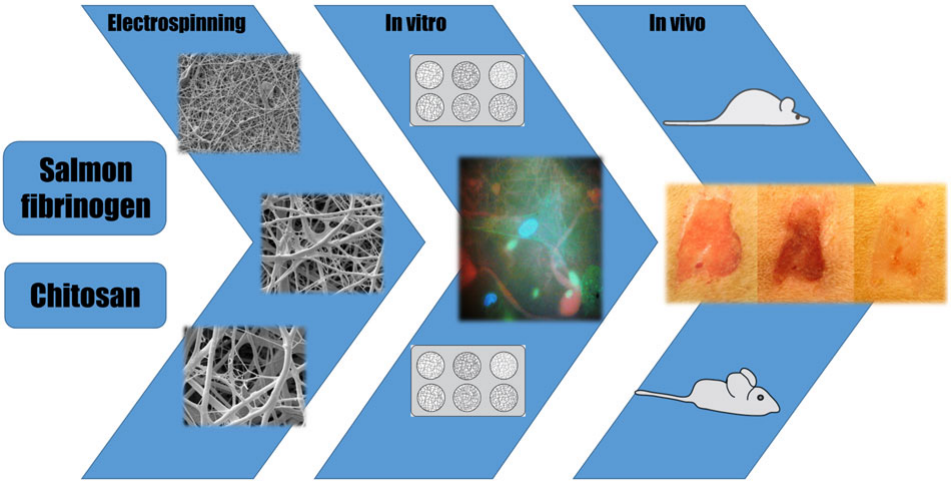

## Introduction

Tissue engineering and wound healing are quickly evolving interdisciplinary areas which have been of great interest for decades. 3D fibrous scaffolds are gaining interest in biomedical settings as platforms for tissue regeneration or cell culture studies, and as support in acute or chronic wound healing and drug delivery systems. In other areas fibrous scaffolds are used as filter material, sensory devices and protective clothing [1, 2]. Their use in regenerative medicine or cellular constructs is justified by their structural similarity to the native extracellular matrix (ECM). These scaffolds can provide chemical and mechanical cues and structural support, which are key elements for cell proliferation and differentiation into functional tissue [3, 4]. The scaffolds should be designed and constructed from nontoxic biodegradable materials to enable the replacement of artificial scaffolds with natural ECM during tissue regeneration. Ideally wound healing products should be safe (low immunogenicity, free from infectious agents like prions and viruses), effective (provide functional tissue recovery) and low cost. Thus, there exists a need for the development of ECM-like materials such as polymer-supported protein filaments that are biocompatible and with good mechanical properties, which also contain sites for cellular adhesion that promote cell growth and function.

Both synthetic and natural polymers are used in tissue engineering and wound healing. Natural polymers like collagen, fibrinogen, chitosan, or structurally similar biocompatible synthetic polymers have been of great interest during the last decades [5]. Among them fibrinogen- and fibrin-based biomaterials are biocompatible and biodegradable and have high affinity for various biological surfaces. Being a naturally occurring physiological material that forms the initial provisional matrix in early stages of wound healing, fibrin scaffolds support angiogenesis and tissue repair. In addition, fibrin naturally contains sites for cellular binding, and has been shown to have excellent cell seeding effects and to promote good tissue development [6]. In addition to the scaffold formed by fibrin itself, platelet-rich fibrin, a fibrin matrix in which platelet cytokines, growth factors, and cells are trapped and may be released can also serve as a resorbable membrane [7, 8].

During the 1980’s, increased awareness of HIV and hepatitis risks from the use of inadequately purified blood and blood products hampered the development of safe and effective human fibrinogen-based haemostatic dressings. Developments in recombinant protein technology and improvements in plasma purification methods have begun to reverse that trend. Still, the structural complexity of fibrinogen makes the production of recombinant proteins impractical or costly. Current sources of fibrinogen used in fibrin-based wound healing products are limited to pooled mammalian blood products. However, issues of contamination and disease transmission are significantly reduced using non-mammalian source of fibrinogen. In particular fibrinogen purified from cold water teleost fish such as Atlantic salmon, has been shown to have many advantages over mammalian fibrin in wound healing contexts in numerous animal studies [6, 9–11].

A number of methods exist for manufacturing ECM-like scaffolds. Electrospinning is a scaffold manufacturing technique that produces nano or microfibers in a continuous manner. Fiber diameter can range from several nanometers to micrometers depending on a number of parameters. Scaffolds made by electrospinning closely mimic natural ECM by possessing properties like high surface area, high porosity and suitable mechanical properties [12].

Many studies have documented the fabrication of electrospun nanofibers that could be potentially useful in regenerative medicine. Also new technologies like Nanospider^TM^ allow nanofibers to be produced on an industrial scale for a number of applications. Hundreds of synthetic and natural polymers have been processed into nanofibers by electrospinning, including fibrinogen, collagen, and chitosan. Beside electrospinning, hydrogel formation by polymerization and crosslinking of polymers is another common technique for scaffold production [13]. Hydrogels are used and studied in numerous wound healing and tissue engineering settings [14, 15]. In addition to their support of cells, they can also be used as carrier of bioactive substances that enhance the growth and function of specific cell types [16]. Although the general principles of electrospinning for the preparation of nanofiber mats are known, every polymer needs well-specified conditions to get the most appropriate product for medical use. Fibrinogen has been most commonly processed into fibers by electrospinning from 1,1,1,3,3,3-hexafluoroisopropanol solutions [17]. Besides being soluble in water, proteins are often soluble in perfluorinated alcohols, such as 1,1,1,3,3,3-hexafluoroisopropanol and 2,2,2-trifluoropropanol.

Electrospun preparations, based solely on fibrinogen have been used for tissue-compatible scaffold generation, but rapid degradation in biological media is a limitation to use pure mammalian fibrinogen scaffolds in wound dressings or 3D cell culture in vitro [18]. Therefore, fibrinogen or other blood clotting factors in a dressing are typically presented in a multilayered setting and often used as lyophilized powders compressed onto a material that serves as the haemostatic dressing support layer [19]. Several combinations of fibrinogen with other components have been described, among which the haemostatic material made from fibrinogen and chitosan, published by Cochrum et al. [20], deserves special attention.

Chitosan is a positively charged polysaccharide composed of *β*(1-4)-linked d-glucosamine monosaccharides with randomly interspersed N-acetylglucosamine. It is made by treating the chitin shells of shrimp and other crustaceans with an alkaline substance such as sodium hydroxide. Chitosan is non-toxic to tissues, and it has been approved by the FDA for use in wound dressings [21]. It is a biodegradable and bioadhesive polymer with bacteriostatic and fungicidal characteristics [22]. Chitosan solutions in concentrated aqueous acetic acid solutions or using trifluoroacetic acid (TFA) as a solvent have been used to form fibrous structures by electrospinning. Electrospinning of chitosan is difficult due to its polycationic character in an acidic aqueous solution. To improve electrospinnability, chitosan has been mixed with other synthetic or natural polymers, or chitosan derivatives have been used including hexanoyl chitosan, quaternized chitosan and N-carboxyethylchitosan [23]. Chen et al. [24] chose the mixture of trifluoroacetic acid and dichloromethane as a suitable solvent for chitosan electrospinning. Though chitosan and collagen–chitosan complex could be dissolved in chosen mixture in their experiments, the formed solution could not be fabricated into nanofibers by electrospinning; only beads and drops formed on the collector.

In order to spin nanofibers from a collagen–chitosan complex, a mixture of hexafluoroisopropanol and trifluoroacetic acid was also tried but collagen became degraded if there was too much TFA in the solution, especially more than 20% [24]. These results show that appropriate selection of a solvent system is a prerequisite for successful electrospinning.

A study by Yuan et al. [25] shows that the creation of unique composites can be limited by intrinsic chemical interactions between the constituent polymers. They observed a polymer precipitate formation when mixing fibrinogen with chitosan. To obtain the fibrinogen/chitosan scaffold they used electrospinning of both components from separate solutions. The capability to electrospin composite polymer/protein fiber is dependent upon finding the optimal solvent system to these chemically distinct solutes and optimizing many other process parameters. There are no current procedures for generation of dressings or matrices of nonwoven chitosan and fibrinogen fibers using easily modifiable one step electrospinning from a blended solution of both components. The polycationic nature of chitosan has limited successful combination with fibrinogen into a single scaffold due to strong electrostatic interactions [25]. In the present study we report development of a procedure to produce a scaffold of salmon fibrinogen and chitosan by electrospinning and characterize the obtained material biologically and physically.

## Materials and methods

### Electrospinning of fibrinogen-chitosan scaffold

Chitosan with molecular weight 160 kDa (DD 90%, Primex Ingredients ASA, Norway), kindly donated by the Faculty of Pharmacy, the University of Helsinki, was used in combination with concentrated trifluoroacetic acid (TFA) (lot 71030, Sigma-Aldrich Laborchemikalien GmbH, Seelze, Germany) or 90% (v/v) aqueous acetic acid (AA) (batch no. 0201/05/04, Polskie odczynniki chemiczne S.A., Gliwice, Poland) solutions. Chitosan solutions in neat TFA and in AA at different concentrations (2–6% (w/v)) were used for electrospinning.

Salmon fibrinogen (lot# 1247, Sea Run Holdings Inc., ME, USA) was dissolved in a mixed solution of 1,1,1,3,3,3-hexafluro-2-propanol (HFP) (Sigma-Aldrich Chemie GmbH, Steinheim, Germany) and TFA (ratio 90:10 (v/v)) at concentrations of 60 mg/ml and 125 mg /ml in HFP/TFA.

In initial studies, chitosan scaffold (CS) and fibrinogen scaffold (FS) were electrospun separately in order to determine the best condition for co-spinning. A 1-ml syringe with a 23- gauge blunt needle was filled either with chitosan in TFA, chitosan in AA or fibrinogen in HFP/TFA solution. The syringe was attached to a syringe pump (KDS 250, KD Scientific Inc., MA, USA). The positive electrode from a high voltage power supply (ES30P-10W/DAM, Gamma High Voltage Research Inc., FL, USA) was connected to the needle. A grounded aluminum foil covered plate was used as the fiber deposition target.

For preparing fibrinogen-chitosan scaffolds (FCS), fibrinogen and chitosan solutions were mixed in a 1:1 (v/v) ratio and stirred for 30 min to obtain a homogenous spinning solution. An optimal condition range was tested for electrospinning according to the literature [26]. Solution feeding rates in the range from 0.1–5 ml/h, voltage from 5–30 kV, and target distance from the electrode between 3–20 cm were tested. Electrospinnability was evaluated by forming a Taylor cone and a stable thin jet. Fibrous structure was visualized by scanning electron microscopy (SEM).

### Scaffold neutralization and sterilization

Neutralization (to maintain the 3D fibrous structure) of electrospun matrices was carried out by using a modified method described by Sangsanoh and Supaphol [27]. Briefly, a suitable size of electrospun scaffold was peeled off from the aluminum foil and submerged in saturated (prepared as 5 M) Na_2_CO_3_ (Reagent, Donetsk, Russian Federation) aqueous solution in a Petri dish. After 1 h incubation with gentle agitation the Na_2_CO_3_ solution was removed and the scaffold was washed 5 × 5 min with deionized water (dH2O). Neutralization was carried out for scaffolds containing chitosan (FCS and CS). For scaffolds containing only fibrinogen (FS), neutralization was omitted, and the scaffolds were only washed with dH2O for 5 × 5 min. For sterilization scaffolds were incubated in 70% (v/v) ethanol for 1 h in a laminar flow hood followed by 3 × 5 min washing with sterile PBS.

### Scanning electron microscopy and image analysis

The morphology of electrospun products was evaluated by a JSM-840 (JEOL, Japan) scanning electron microscope at the Institute of Physics or a Zeiss EVO®15 MA (Germany) instrument at the Institute of Ecology and Earth Sciences, University of Tartu. Samples were mounted on aluminum stubs with carbon tape. Samples containing fibrinogen were sputter coated with 3 nm gold layer in an argon atmosphere prior to microscopy. The measurements were carried out under low vacuum. ImageJ (version 1.50b) software (National Institutes of Health, U.S.) was used to measure the fiber diameters (*n* = 100) from the SEM images [28].

### Gel electrophoresis

40% acrylamide/bisacrylamide solution, 29:1 (3.3% C) was obtained from Bio-Rad (Bio-Rad Laboratories, CA 94547, cat. 161-0146, Control 200002244). Tris-(hydroxymethyl)-aminomethane buffer (Scharlau Chemie S.A., Spain, TR0423) was used to make Tris-HCl electrophoresis solutions. Dodecylsulfate Na-salt (Serva Electrophoresis GmbH, Germany, Lot 18456) was used for 10% SDS aqueous solutions. TEMED (N, N, N’, N’ - Tetramethylenediamine) (Sigma, USA, cat. T9281, Lot 38H0438) and 10% ammonium persulfate (Bio-Rad Laboratories, CA 94804, cat. 161-0700, Lot M6405) aqueous solution were used to initiate acrylamide polymerization. 4-fold sample buffer (0.25 M Tris-HCl, pH – 6.8, 8% SDS, 0.3 M dithiothreitol (Lot 6Y009645, AppliChem GmbH, Germany), 30% glycerol (0361/07/05, POCH SA, Poland) 0.02% bromophenol blue (361901/1397, Fluka Chemie AG) was used as to denature proteins prior to electrophoresis. Molecular markers Precision Plus Protein, All Blue standards (Bio-Rad, cat. 161-0373) and Unstained standards (Bio-Rad, cat. 161-0363) were used to locate proteins of 250, 150, 100, 75, 50, 37, 25, 20, 15 and 10 kDa molecular weight.

The protocol for gel electrophoresis was adapted from our previous work [29] and was modified as described. Protein samples were reduced by adding sample buffer and heated at 95 °C for 4 min. Reduced samples of salmon fibrinogen, salmon fibrin, electrospun salmon fibrinogen and electrospun salmon fibrinogen treated with salmon thrombin (lot #5031, Sea-Run Holdings Inc., ME, USA) were applied on discontinuous SDS-polyacrylamide gels (5% stacking gel and 10% resolving gel) using 1 to 10 µg of protein per 1 cm of gel. Gel electrophoresis was done in a MiniProtean II Electrophoresis Cell apparatus from Bio-Rad (Bio-Rad Laboratories, CA94804, USA) at 150 V. The running buffer was 25 mM Tris buffer (pH 8.3), 0.1% SDS, 192 mM glycine (AppliChem GmbH, Germany, lot 5T009146). Visualization of proteins was done with Coomassie R-250.

### In vitro degradation

The weight loss of the FCS scaffolds (*n* = 4) was determined by the mass change of samples after neutralization of the scaffolds (as described in Scaffold neutralization and sterilization paragraph) and after incubation in Dulbecco’s Modified Eagle’s Medium (DMEM, Sigma-Aldrich, cat. D5796, United Kingdom) at 37^o^C with 5% CO_2_ for 1, 3, and 7 days. At the indicated time point, samples were carefully withdrawn from the medium and rinsed with deionized water and dried for 24 h to remove excess water. The weight loss of the scaffolds was calculated by the following equation: weight loss (%) = (W_0_-W_f_)/W_0_ × 100%. W_0_ was the initial weight of the scaffolds. W_f_ was the weight of the scaffolds at the respective time point.

### Rheometric study

The electropsun sample was hydrated for 24 h before the study by adding deionized water and placed within two parallel plates of an Anton Paar rheometer (MCR502, Anton Paar GmbH, Germany) at the Faculty of Physics, Mathematics and Optometry, University of Latvia. Shear storage and loss moduli (G’ and G”, respectively) were measured as a function of frequency at 5% shear strain and as a function of shear strain at a frequency of 10 Hz. The sample was also statically compressed by decreasing the distance between the plates and the frequency, and strain dependence of G’ and G” of the compressed samples was measured as for the uncompressed sample.

### In vitro cellular compatibility

Neutralized and sterilized electrospun nanofibers of pure chitosan and fibrinogen-chitosan composition were cut into disks of 1.9 mm^2^ to fit into the well of a 24-well tissue culture polystyrene plate (cat. 3526, lot. 11500002, Corning Incorporated, Corning, NY, USA).

Prior to cell seeding, scaffolds were kept in DMEM culture medium (Cat. E15-843, Lot. E84310-0274, PAA Laboratories, Austria) with 10% fetal bovine serum (FBS, PAA Laboratories, Austria) for 1 h at RT. Human dermal fibroblast at their fourth passage obtained from Institute of Cellular and Molecular Biology of the University of Tartu (HF 08/01) were seeded on scaffolds placed on the bottom of tissue culture polystyrene plate wells. Cells were seeded on 9 electrospun chitosan scaffolds and 9 electrospun fibrinogen-chitosan scaffolds. Seeding density was 10 000 cells per well in culture medium (DMEM). One hour after seeding each culture well was gently topped up with 0.4 ml culture medium. This was done to enable cell attachment to scaffold and prevent wash off of cells from scaffolds. The cultures were maintained in an incubator at 37^o^C with 5% CO_2_. Every 2 days the culture medium (0.4 ml) was changed to facilitate optimal growth conditions. On days 2, 4, 8 and 14 two scaffolds of both materials were harvested for proliferation measurements as described further.

Cell proliferation was monitored by the MTS assay—formation of formazan product (CellTiter 96 Aqueous One Solution Cell Proliferation Assay, Promega Corporation, WI, USA). The culture medium was removed, and the cultures were washed with phosphate buffered saline, pH-7.4 (PBS). 400 μl serum free DMEM medium and 80 μl MTS solution were added to each sample well and incubated for 1.5 h at 37^o^C. The obtained colored solution was put into 96-well plates and the samples were analyzed using a microplate reader at 490 nm.

### In vivo wound healing

Ten 21-week-old Wistar rats (males) were obtained from Harlan CPB (The Netherlands) and raised in the Vivarium of the Biomedicum at the University of Tartu (Tartu, Estonia). All animals were kept in the same standard conditions in the same room. Approval for the animal experimentation was obtained from the committee of animal experimentation (Estonian Ministry of Agriculture).

Anesthesia was induced in rats by injection of ketamine (83 mg/kg of Bioketan, lot 9A1514D, Vetoquinol Biowet, Gorzow Wlkp., Poland) and xylasin (6.67 mg/kg of Xylapan, lot 010709D, Vetoquinol Biowet, Gorzow Wlkp., Poland) intraperitoneally. Skin of the back of every animal was prepared for dissection (hairs were shaved, and skin surface was treated with 70 % ethanol). After the drying the skin, split-thickness skin grafts were removed from four sites (approx. area - 1.5 cm × 1.5 cm each) of the rat back using a hand dermatome from E. Weck & Co. Blades (pilling weckprep, cat. No. 450205) used in the dermatome are from TFX Medical Ltd., UK. Adequate pain management was achieved by subcutaneous morphine (Nycomed DAK) injections (5 mg/kg) if necessary.

**Fig. 1:**
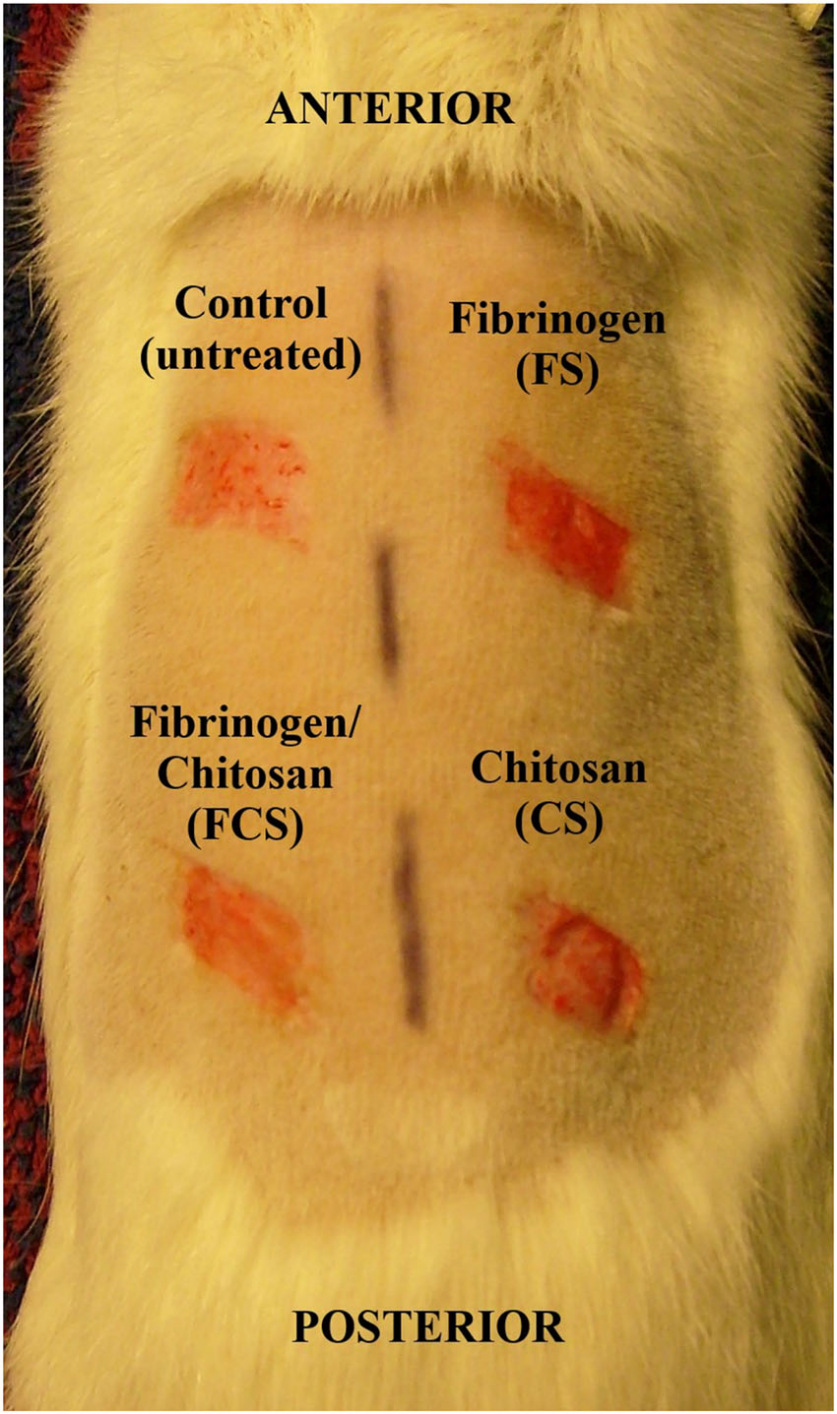
Skin wounds on both sides of the rat dorsal midline after application of treatment regimen. Control – uncovered/untreated wound, FS – covered with fibrinogen scaffold, FCS – covered with fibrinogen-chitosan scaffold, CS – covered with chitosan scaffold

All prepared wound dressing scaffolds were stored at room temperature not more than 3 days before application to the wound area. Prior to application to the wound, the scaffolds were neutralized and sterilized by soaking in 70% ethanol (1 h). Thereafter the scaffolds were repeatedly washed with sterile PBS. Excess PBS was removed by patting the scaffolds with dry gauze, and the scaffold was placed on the wound and held in place with dry gauze for 1 minute. No further wound cover or dressing was used. Scaffolds were used according to the scheme on Fig. 1.

Split-thickness experimental skin wounds testing treated and untreated wounds were compared in a 10-day follow-up period. Wound areas were photographed by digital camera and wound healing (WH) was calculated using formula: WH% = ((A_0_-A_10_)/A_0_) x100 where A_0_ is original wound area and A_10_ is wound area on day 10. Wound areas were measured from photographs with help of ImageJ software.

### Statistical analysis

Data are expressed as means ± standard deviations. Differences among group means for in vivo wound healing were analyzed using one-way Anova analysis with post-hoc Tukey multiple comparison of WH% means. Statistical analysis was performed using the computing environment R [30]. Significance was set at *p* < 0.05.

## Results

### Electrospinning of fibrinogen-chitosan scaffold

**Fig. 2:**
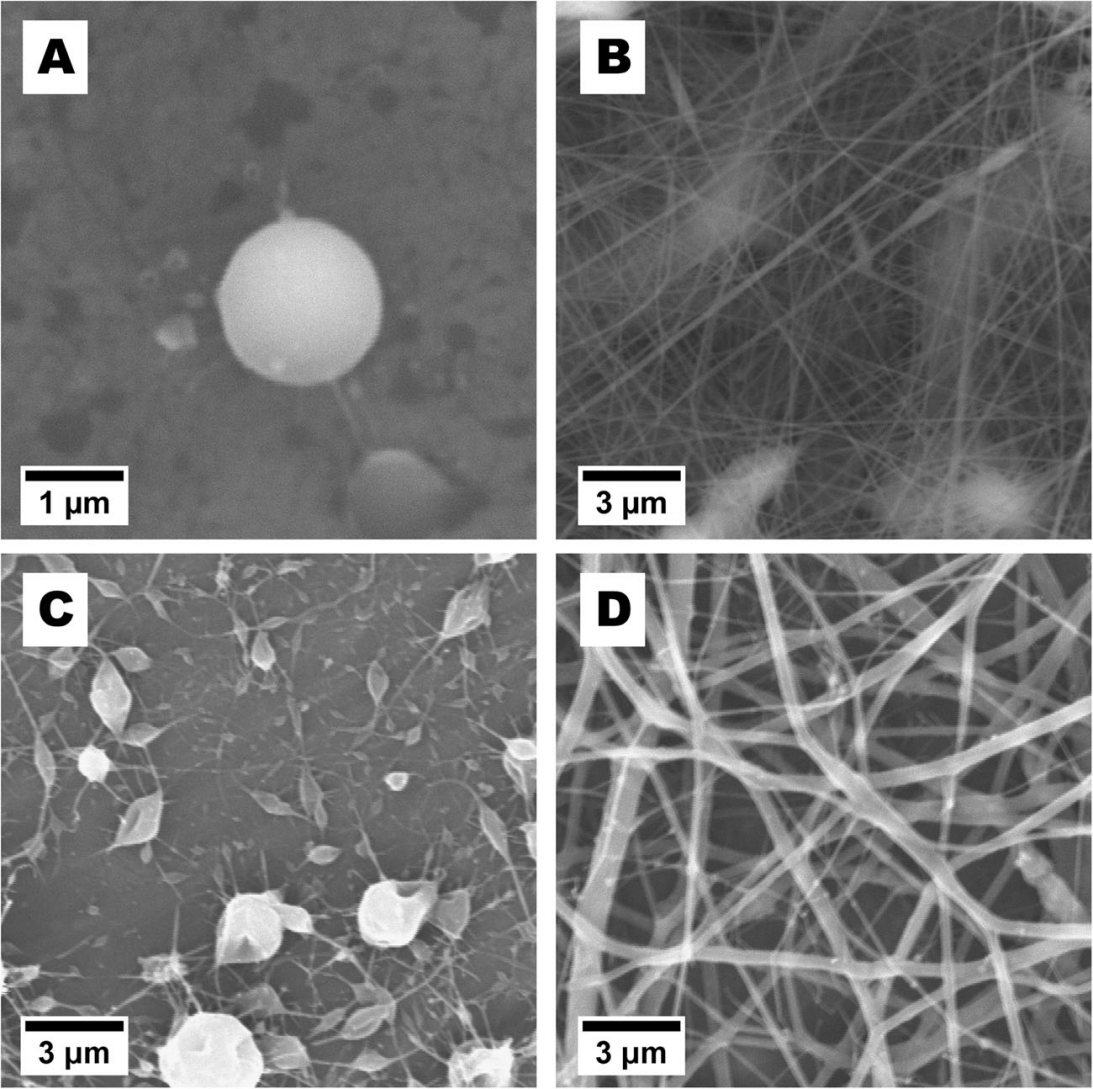
SEM micrographs of **a** electrospun 4% chitosan in 90% aqueous acetic acid solution, **b** electrospun 6% chitosan in TFA, electrospun salmon fibrinogen dissolved in HFP/TFA solution at concentration of 60 mg/ml **c** and 125 mg/ml **d**

To test whether chitosan and salmon fibrinogen are suitable for processing to nanofibrous scaffold, both polymers were electrospun separately at first. Taylor cone forming was observed in all chitosan/acetic acid dilution combinations, but no stable polymer jet was drawn toward the target regardless of changes in electrospinning conditions. No fibrous structure was obtained, only drops of polymer can be seen (Fig. 2a).

In contrast, a uniform nanofibrous mat was formed when chitosan in TFA was electrospun. All chitosan dilutions tested (2%, 3%, 4%, 5 and 6%) showed fibrous structure. In Fig. 2b the structure of electrospun 6% chitosan/TFA solution is shown. A feeding rate 1 ml/h and voltage 1 kV/cm were the best conditions for 6% chitosan/TFA solution electrospinning producing fibers with a mean diameter of 100 nm.

Salmon fibrinogen dissolved in hexafluropropanol (HFP) and trifluoroacetate (TFA) (ratio 90:10) at a concentration of 125 mg/ml in HFP/TFA was best for fiber forming (Fig. 2d). The mean diameter of the obtained fibers was 311 ( ± 155) nm. The range of fiber diameters measured from SEM pictures was from 111 nm to 892 nm. Changes of fibrinogen concentration in the spinning solution changed the fiber morphology. At lower fibrinogen concentrations, heavily beaded fibers and drops were deposited on the target mat (Fig. 2c).

**Fig. 3:**
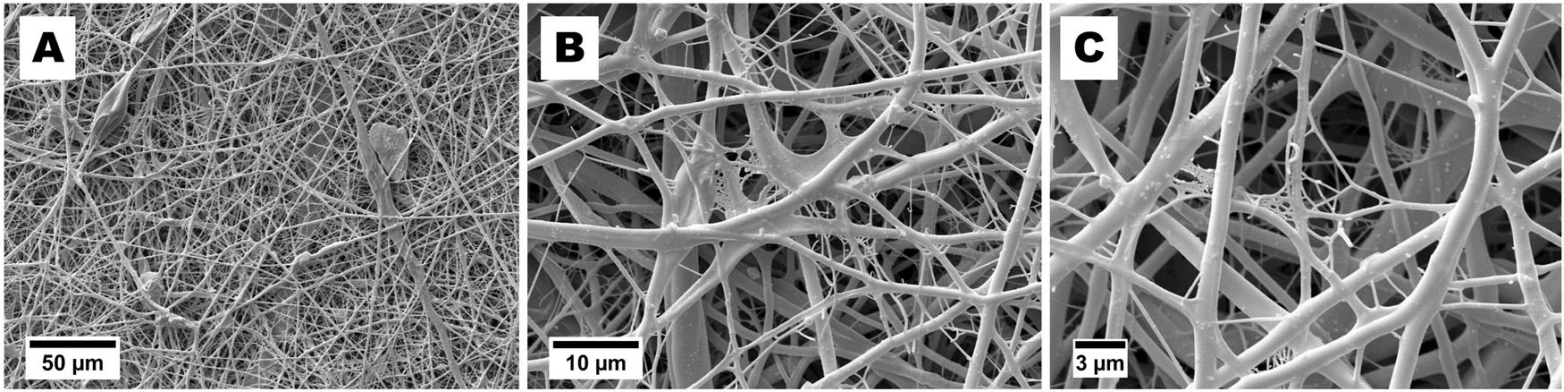
SEM micrographs of fibers electrospun from blended solution of 17.5 mg/ml chitosan and 62.5 mg/ml fibrinogen

For fabrication of nanofiber mats consisting of both chitosan and fibrinogen, separately prepared solutions of 3.5% chitosan in TFA and 125 mg/ml salmon fibrinogen in HFP/TFA were mixed thoroughly and electrospun. After mixing both solutions, the final concentration was 1.75% (17.5 mg/ml) for chitosan and 6.25% (62.5 mg/ml) for fibrinogen. SEM pictures from fibrinogen-chitosan electrospun fibers are shown in Fig. 3. The best results were obtained by using voltage of 11 kV, a feeding rate of 1 ml/h, and a distance from capillary to grounded target of 7 cm. The mean diameter of the fibers obtained was 538 nm with a wide size distribution. The range of fiber diameters measured from SEM pictures was from 165 nm to 2 µm. The thickness (measured with a micrometer) of the fibrinogen-chitosan electrospun scaffold was ca 250 µm.

### Gel electrophoresis

**Fig. 4:**
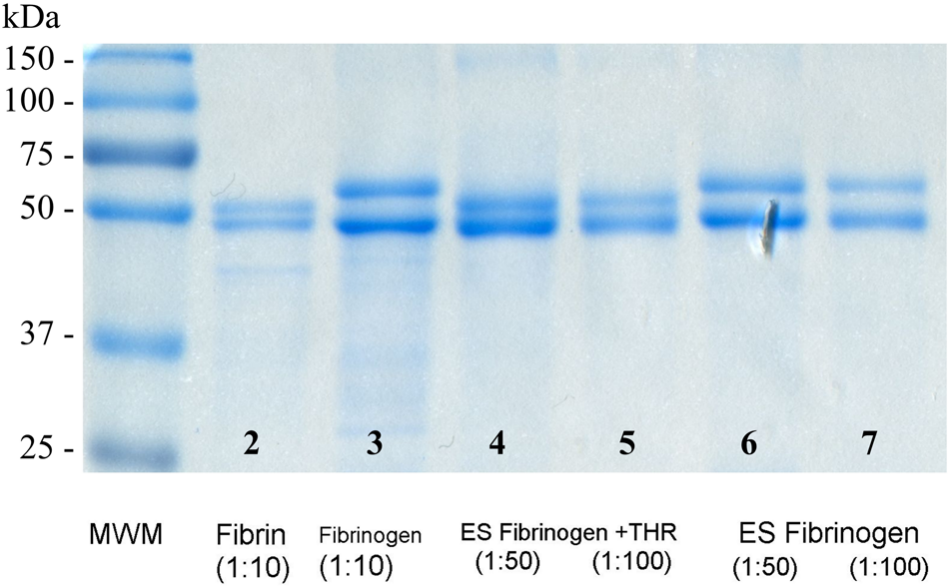
Electrophoretic mobility and apparent molecular weights of reduced peptides of salmon fibrin (column 2), salmon fibrinogen (column 3), ES Fibrinogen + THR – electrospun salmon fibrinogen (treated with salmon thrombin) (columns 4 and 5) and ES Fibrinogen – electrospun salmon fibrinogen (column 6 and 7), MWM – molecular weight markers

To evaluate the changes in molecular mobility of salmon proteins after electrospinning, polyacrylamide gel electrophoresis was done for raw materials and electrospun scaffolds. Figure 4 summarizes the apparent molecular weights of reduced protein samples. Under these conditions two subunits of salmon fibrinogen (Bβ chain ~ 55 kDa and γ-chain ~ 48 kDa) can be seen around 50 kDa molecular weight marker (column 3—“Fibrinogen”). After polymerization of salmon fibrinogen with salmon thrombin, the migration of the β chain to a lower molecular weight can be seen (column 2—“Fibrin”) due to cleavage of fibrinogen peptide B.

Electrospun salmon fibrinogen electrophoresis shows similar protein bands as raw electrospun material around ~ 55 kDa and ~ 48 kDa (column 6 and 7—“ES Fibrinogen”). After incubation of the electrospun scaffold with salmon thrombin, a similar migration of the Bβ chain to an apparent lower molecular weight as seen in the native protein was observed (column 4 and 5—“ES Fibrinogen + THR”). This similarity between native and electrospun proteins suggests that electrospinning of salmon fibrinogen under appropriate conditions does not have a deleterious or degrading effect on the primary structure of the peptides, and therefore the electrospun scaffold maintains the ability to react with exogenously added or endogenously produced thrombin at the wound site.

### In vitro degradation

In vitro degradation of the FCS scaffold was measured after scaffold neutralization and 1, 3 and 7 days after incubation with DMEM. The weight loss after initial neutralization was 52.1 (±1.1%). Neutralization of the FCS scaffold is a prerequisite to maintain its fibrous structure and use in aqueous media. Without neutralization dissolution of chitosan occurs because of the high solubility of salt residues that are formed when chitosan is dissolved in TFA [31].

After further incubation with DMEM weight loss was 53.7% (±1.7%), 55.5% (±0.7%) and 58.3% (±3.8%) after 1, 3 and 7 days accordingly. Our results are in line with other studies where it has been shown that highly deacetylated (DD > 80%) chitosan has low degradation rates [32, 33].

### Rheometry

**Fig. 5:**
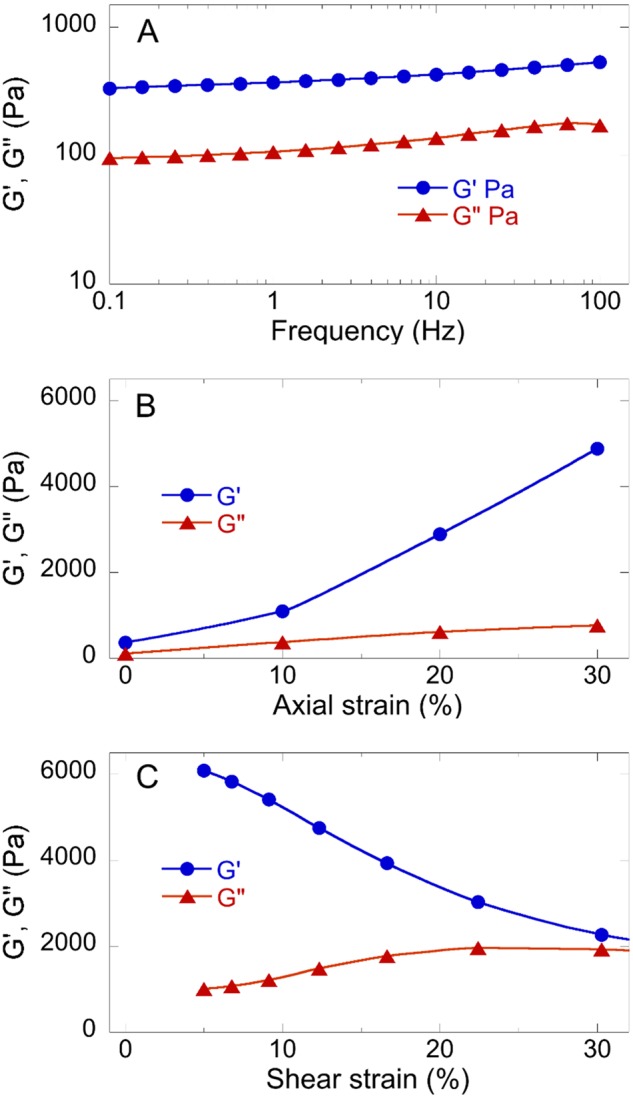
Rheologic response of electrospun fibrinogen-chitosan scaffolds. Shear storage (G’) and loss (G”) moduli of fibrinogen-chitosan scaffolds are functions of **a** frequency at constant 5% shear strain, **b** static axial (compressive) strain at 10 Hz and shear strain 5%, and **c** shear strain at 10 Hz

We measured the viscoelastic response of the combined fibrinogen-chitosan electrospun material to determine if it had mechanical properties appropriate for a wound healing setting. Figure 5a shows that the shear modulus of the hydrated scaffold is on the order of several hundred PA, in the limit of small strain, which is in the range of the stiffness of fibrin at physiological concentrations, and G’ is nearly independent of frequency and significantly larger than G”, characteristic of a viscoelastic solid over a wide range of times. Figure 5b shows that the shear modulus strongly increases when the sample is subjected to uniaxial compression, a feature that is characteristic of many normal soft tissues [34], but not seen in isotropic networks of purified fibrin alone [35], suggesting that the scaffold might have mechanical properties that are closer to the those of the native tissue that those of isolated fibrin networks formed in vitro. Figure 5c shows that in contrast to the stiffening in uniaxial compression, the material does not stiffen with increasing shear strain, again in contrast to the response of purified fibrin gels and suggesting that the elastic properties of the scaffold depend largely on the presence of chitosan in the composite fibers [35].

### In vitro cellular compatibility

**Fig. 6:**
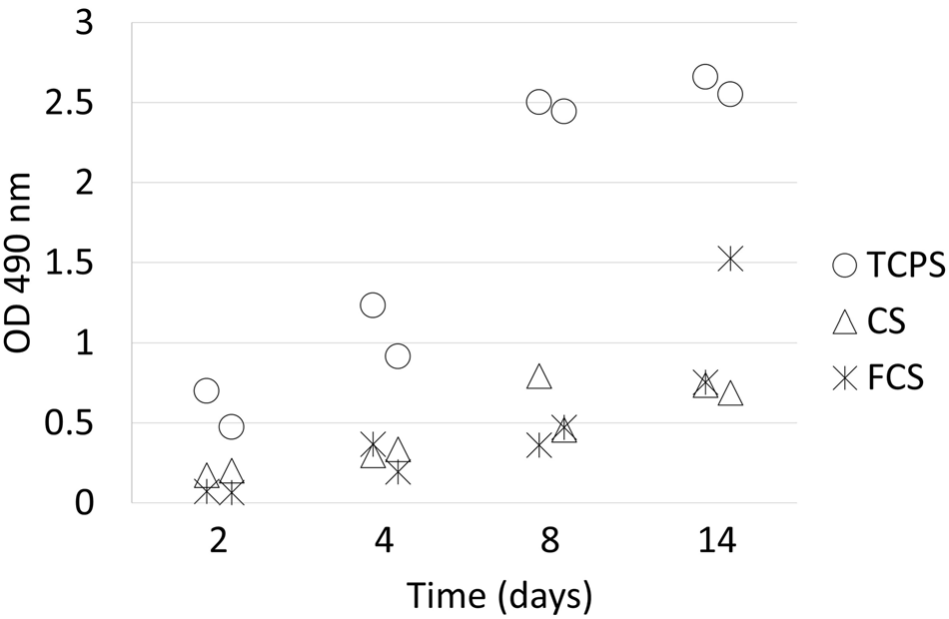
Metabolic activity (formazan dye produced by viable cells) of fibroblasts cultured on chitosan scaffold (CS), fibrinogen-chitosan scaffold (FCS) or tissue culture plate (TCPS) for 14 days as evaluated by MTS assay

Biocompatibility of the fibrinogen-chitosan scaffold (FCS) and chitosan scaffold (CS) was evaluated in vitro by measuring the metabolic activity of fibroblasts cultured on the scaffold for 2–14 days. Human dermal fibroblasts were seeded on the electrospun scaffolds (FCS or CS) or in the wells of tissue culture polystyrene plates (TCPS). On days 2, 4, 8 and 14 two samples from each group were used for the MTS assay. Cells cultured on the TCPS show the highest metabolic activity and proliferation (Fig. 6). The difference between 2D (TCPS) and 3D (electrospun scaffolds) environments is already seen on the 2nd day. Cells grown on 3D scaffolds proliferate at a much lower pace, which is likely related to the softer mechanical environment of the scaffold, since the elastic modulus of the substrate has a strong effect on the proliferation rate of fibroblasts and other cell types [36].

The metabolic activity of the cells increased over time, indicating that the cells were able to proliferate within the scaffolds. Thus, the scaffold obtained by simultaneous electrospinning of the mixture of the solutions of fibrinogen and chitosan is useful for providing a framework for cell growth (attachment) and can be applied in cell culture studies.

### Wound healing

**Fig. 7:**
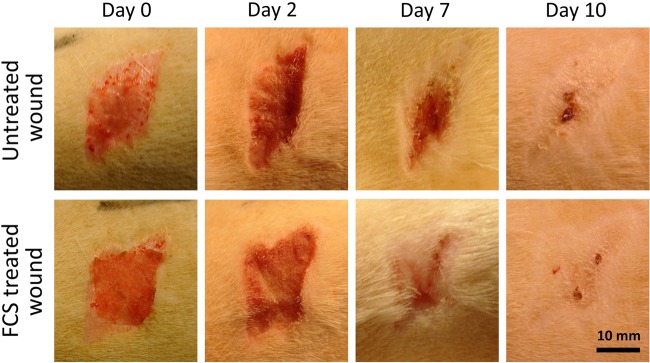
Photographic images of the status of wound healing in untreated (control) and electrospun fibrinogen-chitosan scaffold (FCS) covered group during experiment

Split-thickness skin wounds on 10 rats were compared in a 10-day follow-up period (Fig. 7). Wound healing was calculated using formula: WH% = ((A_0_-A_10_)/A_0_) x 100 where A_0_ is original wound area and A_10_ is wound area on day 10. Results from the wound healing study are summarized in Table 1.

**Table 1:** Wound healing efficiency (WH%, ±*SD*) and size of split-thickness wounds in different groups at the beginning of the experiment (A_0_ mm^2^, ±*SD*) and on day 10 (A_10_ mm^2^, ±*SD*)

	Control	FCS	FS	CS
A_0_ mm^2^ (±*SD*)	291.7 (±42.9)	298.5 (±59.9)	292.0 (±52.6)	273.2 (±30.4)
A10 mm^2^ (±*SD*)	72.8 (±64.7)	29.3 (±13.9)	30.8 (±19.6)	32.4 (±22.0)
WH % (±*SD*)	75.5 (±21.4)	90.0 (±4.2)	89.5 (±6.3)	88.0 (±8.0)

**Fig. 8:**
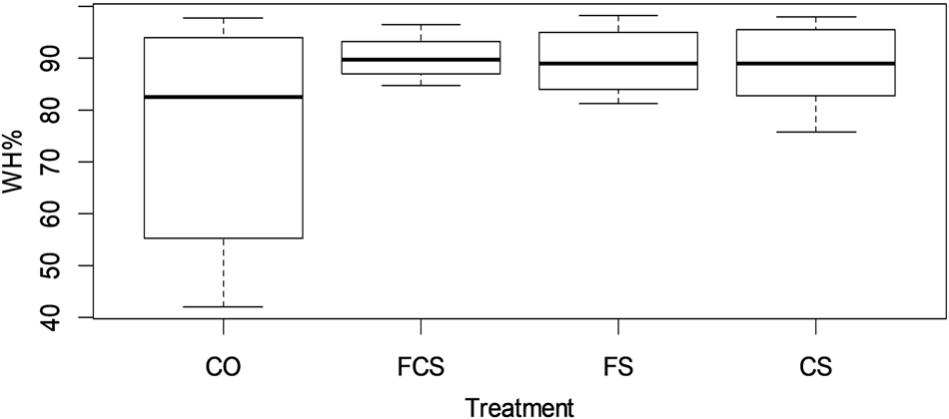
Wound healing efficiency (WH%) at day 10 in control (CO), fibrinogen-chitosan scaffold (FCS), fibrinogen scaffold (FS) and chitosan scaffold (CS) groups

The mean wound healing percent was highest in the FCS treated group (Fig. 8). By comparing wound areas at day 10 the smallest wound area is in the fibrinogen-chitosan scaffold treated group (29.3 mm^2^) followed by wounds treated with fibrinogen scaffolds, chitosan scaffolds, and finally the control (untreated wound) group. Between scaffold treated wounds (FCS, FS and CS) there was only a minor difference in wound healing. One-way Anova analysis showed a statistical difference between treatment regimens. Post-hoc Tukey multiple comparison of WH% means showed a statistically significant difference between FCS and the control group (*p* = 0.049).

## Discussion

In this study we set out to develop a novel scaffold usable for tissue engineering applications in vitro and in vivo. In particular we investigated the optimal scaffold production conditions using chitosan and fibrinogen, materials often explored separately for the development of biologically compatible materials. Another important task was to produce a composite scaffold with optimal properties for cell growth in 3D in vitro cultures.

A number of studies have used chitosan in wound treatment applications, and in tissue engineering applications such as formation of cartilage tissue, bone substitutes, support of respiratory epithelial cells for tissue-engineered trachea or in nerve cell attachment and proliferation experiments [37]. It has been reported that chitosan acts as a chemo-attractant to macrophages and neutrophils during wound healing. Chitosan accelerates the development of tensile strength in wounds by speeding the fibroblastic synthesis of collagen in the initial phase of wound healing. Chitosan has an analogous structure with glycosaminoglycans, which are major components of native ECM. However, based on an in vitro study protocol, chitosan scaffolds alone did not support human dermal fibroblast (HDF) attachment [38]. Chitosan has been shown to have anti-inflammatory and anti-microbial properties, which could be useful in wound healing materials, and has been tested in different forms (powder, hydrogel, sponge materials, fibrous dressing) and wound settings (acute, burn, infected wounds). Although chitosan dressings have also been developed to address hemorrhage problems, they are not always effective in controlling bleeding [37].

Azad et al. [39] evaluated meshed and non-meshed chitosan membranes for wound dressings. Meshed chitosan membranes promoted faster healing, better organization of the repaired tissue including re-epithelialization, and a cosmetically acceptable outcome. This result confirms the importance of porosity when designing scaffolds for wound healing [40]. In another study, Kratz et al. [41], the effect of chitosan-heparin membranes on wound healing in human skin was evaluated. Chitosan-heparin complexes showed faster and more complete re-epithelialization after 12 days, compared to the donor untreated sides where an incomplete re-epithelialization was observed even after 15 days.

Chitosan dressings have also been developed to control bleeding and infection, but they are not always effective. In a study by Kheirabadi and colleagues [42], a chitosan dressing consistently failed to secure haemostasis within 2 h after application, which may indicate a lack of adhesion sites in chitosan dressings.

Combination of chitosan with blood proteins to develop scaffolds for tissue regeneration is a relatively new area of research. Among proteins added to chitosan, fibrinogen (or fibrin) deserves special attention as a coagulation protein. Salmon fibrinogen, which produces less inflammatory response than mammalian fibrin [9, 43–45] has been explored in the present study. As another new development we have produced for the first time a fibrinogen-chitosan complex by careful choice of physical and chemical conditions in the electrospinning procedure, in the form of a biocompatible material mimicking the structure of the native ECM.

The resulting matrix is heterogeneous in fiber diameter (from 165 nm to 2 µm) which resembles the natural ECM (interstitial matrix) structure where larger fibrils are interspersed with much smaller fibers. Based on electrophoretic mobility it is seen that the non-aqueous solvents and electrospinning do not have a deleterious effect on the protein, and the scaffold maintains the ability to react with thrombin. It has been shown by others that use of TFA in electrospinning can lead to protein decomposition. Chen et al. investigated collagen–chitosan electrospinning and found that the strong acidity of TFA is a possible cause of collagen degradation [46]. It has been shown also by others that in the presence of an increasing concentration of TFA (already in mM concentrations) proteins tend to undergo extensive unfolding [47].

Since our scaffold supports cell growth in 3D cultures, as evaluated by human dermal fibroblast cultures, this material has potential for large-scale use in bioengineering. 3D models can better replicate intrinsic physiological conditions and in vivo cellular responses to external stimuli compared to a 2D monolayer [48]. The fibrinogen used in this study contains fibronectin and fibrinogen fragments as copurifying impurities providing potentially better adhesion by binding to cell surface integrins [9]. Copurifying salmon plasma fibronectin constitutes 3% of fibrinogen preparations unless it is specifically removed. Fibronectin enhances cell adhesion and spreading and affects the routes of cell migration both in vivo and in vitro. It has been shown that cells in the fibronectin-functionalized scaffold exhibit different aggregation patterns that could be related to the distinct mRNA expression levels of cell adhesion-related genes [49].

Carlisle et al. [50] analyzed the mechanical properties of a single electrospun fibrinogen nanofiber, using a combined atomic force/fluorescence microscopic technique, and showed that a fibrinogen nanofiber has a high extensibility. High-extensibility fiber scaffolds have been found to be more widely applicable for in vitro and in vivo tissue engineering due to their flexibility. The rheometric data show that the mechanical properties of fibrinogen-chitosan electropsun scaffolds have important similarities to those of native ECM-containing tissues. Figure 5 shows that their elastic shear modulus (G’) is significantly larger than the loss modulus G” over a large frequency scale, but that G” is within an order of magnitude of G’, consistent with the values of many soft tissues [34]. Moreover, the mechanical responses of these scaffolds are highly non-linear, with the shear modulus increasing strongly with compression. This stiffening of the scaffolds with increasing compression is not seen in simpler hydrogels such as PEG-based scaffolds made by flexible polymers and shares some features with native ECM and soft tissues. Fibrous networks formed by fibrin and other rigid or semiflexible polymers such as collagen have complex rheological response with a very small and often technically inaccessible linear elastic range [35]. Therefore, measurements of shear moduli at shear and compressive strains in the non-linear range, characteristic of physiological deformations [34], are important for comparing the properties of bioengineered materials and the soft tissues they are designed to mimic.

Some data are available about the effect of fibrinogen-chitosan complex preparations on wound healing, although these materials have not yet been produced by electrospinning. In one study [51] fibrin-chitosan composite films were evaluated on canine cutaneous wounds by observing the presence of discharges, granulation tissue, scar formation, wound contraction, wound healing, and other complications. Fibrin-chitosan treated wounds showed neither hemorrhage nor adverse inflammatory reaction by the host tissue, which might be due to sealant and hemostatic activity of the biocasing. It was concluded that fibrin/chitosan treated wounds showed early healing and that the biomaterial can be safely be applied for cutaneous wound healing in dogs. In a study by Sudheesh et al. [52] chitosan and fibrin bandages, consisting of fibrin nanoparticles impregnated in a chitosan hydrogel, were tested for wound healing. These bandages showed high cell viability, cell adhesion, and enhanced blood clotting when compared to chitosan-only bandages. In rats, partial thickness skin wounds treated with the bandages showed 98% wound closure. The wounds treated with either chitosan-only bandages and the controls (bare wounds) showed only 70% wound closure. It was proposed that the presence of fibrin nanoparticles, in conjunction with the biodegradation of chitosan matrix, might produce an additive effect on wound healing [53].

## Conclusions

No dressings or matrices consisting entirely of separate chitosan and fibrinogen nano or microfibers (uniformly distributed within the matrix) made by one step have previously been described. This combination, presented in our study, exploits the characteristics of fibrinogen by acting as a haemostatic agent that subsequently supports angiogenesis and tissue repair, as well as providing better adhesion properties than chitosan alone. Furthermore, the combination of chitosan with fibrinogen in electrospun scaffolds improves their mechanical properties, which are poor in electrospun fibrinogen alone.

In vitro cell culture and in vivo wound healing studies show that use of salmon fibrinogen and chitosan in combination has a better outcome than that of either substance separately. However, further studies with cell lines of other origins (e.g. neurons [54] and stem cells [55, 56]) are needed to fully reveal the specific benefits of salmon fibrinogen-chitosan scaffolds.

In the present study we were able to develop a method for incorporating chitosan and fibrinogen into one preparation that might be usable for the medicinal applications such as haemostasis, wound healing, or producing substrates for 3D cell cultures.
